# Web Resources for Pharmacogenomics

**DOI:** 10.1016/j.gpb.2015.01.002

**Published:** 2015-02-19

**Authors:** Guoqing Zhang, Yunsheng Zhang, Yunchao Ling, Jia Jia

**Affiliations:** Shanghai Center for Bioinformation Technology, Shanghai 201203, China

**Keywords:** Pharmacogenomics, Pharmacogenetics, Drug response, Adverse reaction

## Abstract

Pharmacogenomics is the study of the impact of genetic variations or genotypes of individuals on their drug response or drug metabolism. Compared to traditional genomics research, pharmacogenomic research is more closely related to clinical practice. Pharmacogenomic discoveries may effectively assist clinicians and healthcare providers in determining the right drugs and proper dose for each patient, which can help avoid side effects or adverse reactions, and improve the drug therapy. Currently, pharmacogenomic approaches have proven their utility when it comes to the use of cardiovascular drugs, antineoplastic drugs, aromatase inhibitors, and agents used for infectious diseases. The rapid innovation in sequencing technology and genome-wide association studies has led to the development of numerous data resources and dramatically changed the landscape of pharmacogenomic research. Here we describe some of these web resources along with their names, web links, main contents, and our ratings.

## Introduction

Pharmacogenomics, closely associated with pharmacogenetics, is the study of genetic variations that influence the response of individuals to drug treatment [1], [2]. The major contribution and aim of pharmacogenomics is to understand the relationship between the genetic variations among individuals of different genotypes and their reactions to drugs. This information can be employed to determine the specific medications to be used during the treatment of an individual patient, and thus, to enable a personalized approach to healthcare. It is well known that a medication with proven efficacy in a large patient population may fail to show clinical benefits, but cause various adverse drug reactions (ADRs) instead, and even lead to death in a small group of patients carrying certain genetic variations [3].

For the past five decades, genomic and clinical scientists have been studying samples taken from specific diseases and drug-response phenotypes to develop a more precise method of using medication and help prevent some of the avoidable ADRs. Some research discoveries have already shown substantial clinical benefits. For patients with cardiovascular disease, warfarin may be the most widely prescribed oral anticoagulant; however, warfarin can induce serious adverse responses like hemorrhage and undesired coagulation that may often lead to emergency room visits [4]. Pharmacogenomic and genome-wide association studies have dramatically changed this situation by identifying the *CYP2C9*, *VKORC1*, and *CYP4F2* genotypes that influence the final warfarin dose needed for effective anticoagulation [5]. The Food and Drug Administration (FDA) of the United States (US) revised the drug label of warfarin in 2010, and since then, patients’ genotypes have been taken into consideration when prescribing warfarin [6]. Cancer pharmacogenomics is more complicated since it must take into account both the germline and somatic genomes of the tumor [7]. After sequencing a large amount of kinase genes that exhibit somatic variations in tumors, pharmacogenetic research identified a specific inhibitor, vemurafenib, which can effectively prolong the survival of patients who carry a somatic mutation in *BRAF* [8]. Other researchers have shown that variations in the germline genomes of patients are clinically relevant and may affect anti-cancer drug therapy [9], [10]. Empowered by the advances in sequencing technology and genome-wide association studies, pharmacogenomic scientists are now capable of gathering larger and more precise amounts of genomic and clinical data. Thus, additional scientific literature, popular press articles, and useful web resources related to pharmacogenomic studies have been published in recent years. Some of these resources associated with applications are now, or will be, an important reference in pharmacogenomic research and clinical practice. Here we describe some of these web resources, including their names, web links, main contents, and an extra rating column (Table 1). We rated these web resources based on several factors including their relations to pharmacogenomics, their data sizes, the number and usefulness of the applications provided on the website, and the overall user experience. We hope these web resource will extend our understanding of pharmacogenomics and drug mechanisms.

**Table 1:** Major web resources for pharmacogenomics

**Name**	**Link**	**Main features**	**Rating**	**Refs.**
PharmGKB	http://www.pharmgkb.org/	NLP and manual curation variants, associations between variants and drugs, drug-centered pathways, genotype-based pharmacogenomic summaries	★★★★★	[11]
CPIC	http://www.pharmgkb.org/page/cpic/	Detailed gene/drug clinical practice guidelines, drug dosing guidelines	★★★★☆	
DrugBank	http://www.drugbank.ca/	Drug-based information, drug pharmacogenomics, drug/food interaction, metabolic enzymes, QSAR data, ADME data	★★★★★	[12]
SCAN	http://www.scandb.org/	SNP annotation, GWAS analysis tool	★★☆☆☆	[13]
PACdb	http://www.pacdb.org/	Pharmacology-related information including genotypes, gene expressions, and pharmacological data obtained via LCL	★★★☆☆	[14]
Human Cytochrome P450 Allele Nomenclature database	http://www.cypalleles.ki.se/	CYP450 alleles, CYP450 isoforms, relationship between genotype and phenotype	★★★★☆	
Cytochrome P450 Drug Interaction Table	http://medicine.iupui.edu/clinpharm/ddis/clinical-table/	Drug and CYP450 isoform interaction	★★★★☆	
FDA’s pharmacogenetic website	http://www.fda.gov/drugs/scienceresearch/researchareas/pharmacogenetics/ucm083378.html	Clinical response and drug exposure variability, dosing recommendation according to genotypes, drug mechanisms, germline or somatic gene variant biomarkers	★★★★★	

*Note:* The web resources are rated based on several factors including the resources’ relations to pharmacogenomics, their data sizes, the number and usefulness of the applications provided on the website, and the overall user experience. NLP, natural language processing; QSAR, quantitative structure activity relationship; ADME, absorption, distribution, metabolism, and excretion; LCL, lymphoblastoid cell line; CYP450, cytochrome P450.

## PharmGKB

The Pharmacogenomics Knowledgebase (PharmGKB) is a database of genetic variations, annotations, drug pathways, and their relationship with drug response [11]. It is a project managed by Stanford University and supported by the NIH/the National Institute of General Medical Sciences (NIGMS). PharmGKB aims to help researchers to understand how genetic variations in different individuals can affect drug reactions. Information in the PharmGKB database is mainly derived from the scientific literature, and is properly stored and displayed at different levels. The pharmacogenomic studies are analyzed using natural language processing (NLP) technology with manual curation of the content. To facilitate the analysis of results, clinically-relevant information including annotations of gene variants, pathways related to drugs, and some very important pharmacogene (VIP) summaries are provided. Each annotation is supported by one or more published articles that describe the variant or genotype. Some pre-clinical data derived from *in vitro* experiments and animal studies are also collected in PharmGKB. *In vitro* and *in vivo* animal data may not be as clinically significant as drug testing in humans, but it may hint at the possible, or biologically plausible, variations in drug response due to genetic makeup. Based on these comprehensive genomic variation annotations, PharmGKB not only summarizes the complicated relationship between genotype and drug response, but also makes a deeper clinical interpretation of varied drug responses as well as ADRs within individuals possible. Additionally, PharmGKB also integrates information from the Clinical Pharmacogenetics Implementation Consortium (CPIC) to provide drug-dosing guidelines according to personal genotype. Currently, PharmGKB comprises information on over 26,000 genes and over 3000 drugs, including 54 VIP summaries and 113 pharmacokinetic and pharmacodynamic pathways. PharmGKB is updated periodically in response to newly-published papers, and presents all its data such as variations, annotations, summary information, and guidelines on its website. PharmGKB is well known and is the preeminent resource for translational researchers and clinical doctors to implement pharmacogenomics information in their research or clinical practice.

## CPIC

The CPIC was formed in 2009 as a shared project of both the Pharmacogenomics Research Network (PRN) and PharmGKB. The CPIC aims to provide detailed gene/drug clinical practice guidelines to promote the integration of pharmacogenetic research into clinical practice. The CPIC collects all levels of scientific evidence, from biological research to clinical studies, and evaluates and incorporates this scientific evidence into the guidelines. Rather than defining the indications for testing, these CPIC guidelines will help the clinicians understand how a genetic test can be used to optimize drug therapy. A CPIC guideline is a comprehensive system of evidence linking genotypes with phenotypes including the rules of assigning phenotypes to genotypes, the rules of prescription according to genotypes or phenotypes, and the strength of the evidence. To date, the CPIC has collected 174 gene/drug pairs of all CPIC levels, including 63 genes and 132 drugs. All CPIC guidelines are validated by peer review, updated periodically, and freely available via the CPIC website.

## DrugBank

Unlike PharmGKB, which focuses on drug reactions according to human genomic variations or specific genotypes, the aim of DrugBank is to build a comprehensive resource on drugs including their pharmacological and biochemical information, mechanisms, and targets [12]. The first version of DrugBank was released in 2006 and provided data only on selected FDA-approved drugs and their targets. With the development and upgrading of the database, more information is now available including, but not limited to, pharmacological, pharmacogenomic and molecular biological, drug-food and drug-drug interaction, metabolic enzymes, and quantitative structure activity relationships (QSAR) data as well as other drug-related data such as absorption, distribution, metabolism, and excretion (ADME) profiles. The basic drug information such as drug names, structures, salt-forms, targets and actions are still being expanded upon and updated. For the current release, DrugBank 4.0, more than 1200 drug metabolites and more than 1300 drug metabolism reactions, in addition to dozens of drug metabolism pathways, have also been added to the database. In comparison to PharmGKB, DrugBank is a comprehensive database of drugs that can facilitate *in silico* drug design, drug target discovery, drug screening or docking, interaction prediction, metabolism prediction, and pharmaceutical education.

## SCAN and PACdb

SCAN and PACdb are two pharmacogenomic databases developed and published by the Pharmacogenomics of Anticancer Agents Research group (PAAR). PAAR is a research group focusing on pharmacokinetics and pharmacodynamics, which aims to discover and validate functional germline polymorphisms relevant to anticancer agents, especially those used in the treatment of solid tumors in adults.

SCAN is designed to collect, annotate, and present the relationship between genotype and gene expression. It is a large-scale genetic and genomic database containing single nucleotide polymorphism (SNP) and copy number variation (CNV) annotations along with a web interface, a set of methods and algorithms, and some data mining tools [13]. Data that are stored in SCAN includes physical-based SNP annotations that are categorized according to gene coordination or linkage disequilibrium (LD) patterns, and functional SNP annotations that are classified by their effects on expression levels. However, the current release of SCAN only collects data from HapMap cell lines. In addition to the SNP querying system, SCAN also provides some follow-up analysis applications such as a genome-wide association study (GWAS) analysis tool. This tool takes advantage of the tens of thousands of correlations generated in GWAS, thus helping users of SCAN to connect their SNP query results with their association to diseases. Therefore, the main use of SCAN is to query the database, to perform an analysis using the attached applications and methods, and finally, to download the result files including SNPs, genes, and GWAS traits.

PACdb is a pharmacogenetics-cell line database collecting pharmacology-related information including genotypes, gene expression, and pharmacological data gathered via lymphoblastoid cell lines (LCL) [14]. Data in PACdb include microarray expression data from 90 Yoruba in Ibadan, Nigeria (YRI) and 90 Utah residents with ancestry from northern and western Europe (CEU) LCLs, and some microRNA data from 60 YRI and 60 CEU. PACdb set a model on human whole genome to connect gene expression and genotypes with cytotoxicity in LCL. PACdb may serve as an extension to SCAN, allowing users to query for genotypes or gene expression data with their relative drug responses.

## Human cytochrome P450 databases

Human cytochrome proteins are located either in the inner membrane of mitochondria or in the endoplasmic reticulum of cells [15]. Cytochrome P450 (CYP450) enzymes are present in most tissues of the body, and they are the major enzymes involved in drug metabolism, accounting for about 75% of the total metabolism [16]. The changes in CYP450 enzyme activity may affect the metabolism of various drugs; hence, they are a major modulator of adverse drug interactions. The Human Cytochrome P450 Allele Nomenclature website is a useful database that properly stores the CYP450 alleles and the relationship between CYP450 genotypes and phenotypes. It is also a CYP450 official and unified allele designation system. Using the database, a user may easily find a CYP450 isoform along with the nucleotide changes, trivial name, effect, *in vivo* or *in vitro* enzyme activity, and the reference published papers that support this record. The Cytochrome P450 Drug Interaction Table is also a resource that contains a list of drugs under the designation of CYP450 isoforms. These resources, which suggest that genetic variations play a role in every step of drug metabolism, will help researchers, clinicians, and health care providers during their pharmacogenomic research and clinical practice.

## The pharmacogenetic website of the US FDA

Pharmacogenomic research is an important part of genomic research that benefits clinical practice since the patients’ genotypes may help the clinicians to make better drug choices, avoid ADRs, or to prescribe the right dose. Therefore, the US FDA set up a specific pharmacogenetic website to label drugs with genomic biomarkers and other information about drug dosing or ADRs. According to the website, the factors affecting clinical response and drug exposure variability include adverse reaction risks, recommended dosing according to genotypes, drug targets, disposition genes, and drug mechanisms. The suggested biomarkers include somatic or germline gene variants, expression changes, functional deficiencies, and abnormalities in chromosomes. The website consists of a table of drugs and pharmacogenomic biomarker labels approved by the FDA. Currently, 87 out of 158 drugs recorded in the table are labeled with different kinds of pharmacogenomic information. However, the website only collects human genetic biomarkers while disregarding bacterial or viral biomarkers. More information and links on the drugs, such as their usage and indications, administration and dosage, or some box warnings, can also be found in the rest of the columns of the table for further understanding of the drug and its pharmacogenomic properties.

## Conclusions

The development of sequencing technology and GWAS has increased the feasibility and accuracy of pharmacogenomic study, and led to the publication of a good deal of pharmacogenetic and pharmacogenomic literature. Scientists and clinicians are now capable of using the web resources mentioned above (Table 1), along with some useful applications, to improve several aspects of drug therapy like drug selection, administration, and avoidance of ADRs. Nonetheless, challenges still exist in learning how to most effectively use the massive amount of sequencing or laboratory findings to promote the understanding of drug mechanisms. Resolving this issue will be the focus of pharmacogenomic researchers, clinical doctors, computer scientists, data-mining engineers, and database administrators in the near future.

## Competing interests

The authors declare that there are no conflicts of interest.
